# Supplementary material for: BNT162b2 COVID-19 Vaccines in Children, Adolescents and Young Adults with Cancer—A 1-Year Follow-Up
Source: Vaccines (Basel). 2023 May 16;11(5):989. doi: 10.3390/vaccines11050989 (PMC10224057; doi:10.3390/vaccines11050989)
Supplement: Supplementary file 1 [file vaccines-11-00989-s001.zip › vaccines-2353363-supplementary.pdf]

Table S1. Serology per month post vaccination.

	Median (BAU/ml)	Q1 (BAU/ml)	Q3 (BAU/ml)	Covid-19 infection	N
Month 0 (day 0)	26	26	26	0	20
Month 1 (day 1 to 30)	173	26	498	2	21
Month 2 (day 31 to 60)	204	44	2252	0	12
Month 3 (day 61 to 90)	1778	1379	3099	0	7
Month 4 (day 91 to 120)	431	166	1578	0	10
Month 5 (day 121 to 150)	904	150	1720	2	8
Month 6 (day 151 to 180)	895	367	3219	1	5
Month 7 (day 181 to 210)	376	82	708	0	11
Month 8 (day 211 to 240)	88	88	88	0	1
Month 9 (day 241 to 270)	216	162	2088	1	4
Month 10 (day 271 to 300)	3349	816	6007	0	4
Month 11 (day 301 to 330)	1198	668	7419	1	3
Month 12 (day 331 to 360)	6437	3524	7058	0	3
> Month 12 (> day 361)	5878	696	7138	0	8

Table S2. Serology per month post vaccination (exclusion of patients with COVID-19 infection).

	Median (BAU/ml)	Q1 (BAU/ml)	Q3 (BAU/ml)	N
Month 0 (day 0)	26	26	25,6	19
Month 1 (day 1 to 30)	220	26	679,7	19
Month 2 (day 31 to 60)	1383	217	2997,6	8
Month 3 (day 61 to 90)	1603	1304	3481	5
Month 4 (day 91 to 120)	500	250	1725,9	10
Month 5 (day 121 to 150)	976	203	1749,1	6
Month 6 (day 151 to 180)	2057	763	3849	4
Month 7 (day 181 to 210)	624	306	877	9
Month 8 (day 211 to 240)	88	88	88	1
Month 9 (day 241 to 270)	216	162	2088	4
Month 10 (day 271 to 300)	3349	816	6007	4
Month 11 (day 301 to 330)	1888	1013	2764	2
Month 12 (day 331 to 360)	3524	2068	4980	2
> Month 12 (> day 361)	5507	553	6836	7

Table S3. Classification of the severity of COVID-19 (WHO classification).

Severity of COVID-19	
Asymptomatic or presymptomatic infection	Individuals who test positive for SARS-CoV-2 using a virologic test (i.e., a nucleic acid amplification test [NAAT] or an antigen test) but who have no symptoms that are consistent with COVID-19.
Mild illness	Individuals who have any of the various signs and symptoms of COVID-19 (e.g., fever, cough, sore throat, malaise, headache, muscle pain, nausea, vomiting, diarrhea, loss of taste and smell) but who do not have shortness of breath or abnormal chest imaging.
Moderate illness	Individuals who show evidence of lower respiratory disease during clinical assessment or imaging and who have an oxygen saturation measured by pulse oximetry (SpO2) ≥94% on room air at sea level.
Severe illness	Individuals who have SpO2 <94% on room air at sea level, a ratio of arterial partial pressure of oxygen to fraction of inspired oxygen (PaO2/FiO2) <300 mm Hg, a respiratory rate >30 breaths/min, or lung infiltrates >50%.
Critical illness	Individuals who have respiratory failure, septic shock, and/or multiple organ dysfunction.
